# Supplementary material for: The fluorination effect of fluoroamphiphiles in cytosolic protein delivery
Source: Nat Commun. 2018 Apr 10;9:1377. doi: 10.1038/s41467-018-03779-8 (PMC5893556; doi:10.1038/s41467-018-03779-8)
Supplement: Supplementary file 1 — Supplementary Information(PDF 5253 kb) [file 41467_2018_3779_MOESM1_ESM.pdf]

# The fluorination effect of fluoroamphiphiles in cytosolic protein delivery

**Zhang et al.**

## Supplementary Figures

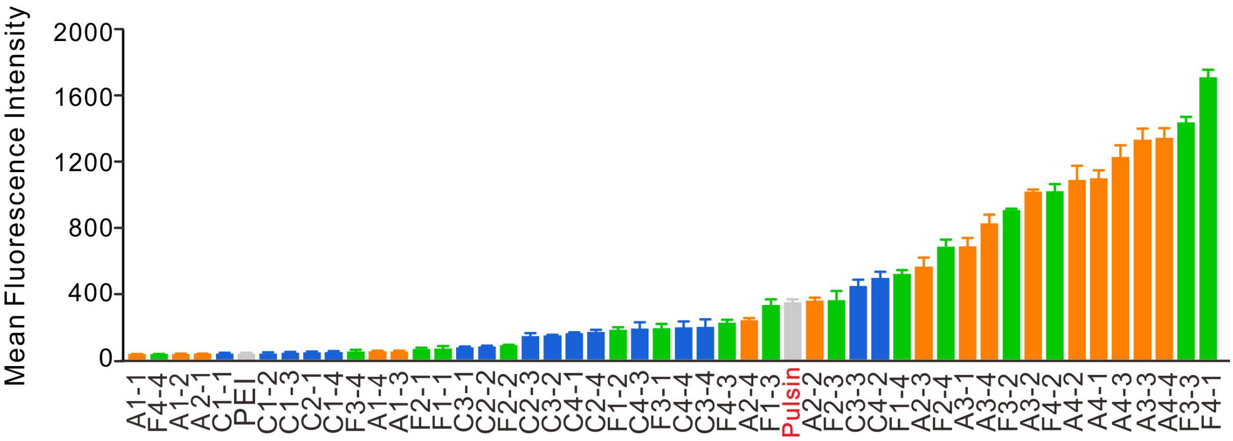


**Supplementary Fig. 1.** Efficiacies of amphiphiles in the delivery of BSA-FITC. Mean fluorescence intensity of HeLa cells transfected with amphiphile/BSA-FITC nanocomplexes for 4 h at their optimal doses analyzed by flow cytometry. Data are presented as the mean ± s.e.m. (n=3).

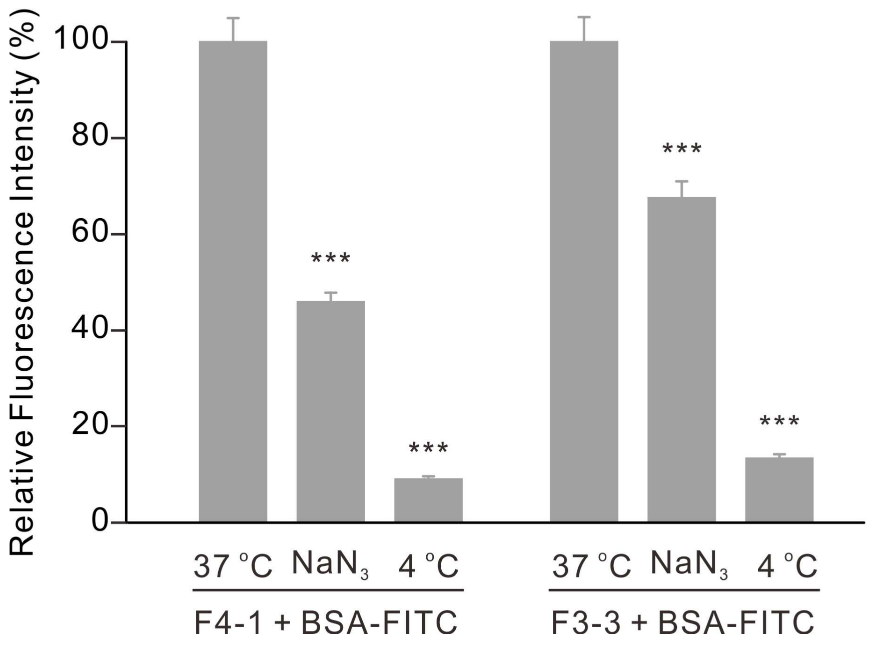


**Supplementary Fig. 2.** Endocytosis of fluoroamphiphile/BSA-FITC complexes. Relative fluorescence from HeLa cells treated with F4-1/BSA-FITC and F3-3/BSA-FITC complexes under various conditions. 4 μg BSA-FITC (0.3 μM) was complexed with 2 μg F4-1 and 2.5 μg F3-3, respectively. The concentration of $NaN_3$ is 100 mM. The cells were treated with 0.2 mg/mL trypan blue to quench the physically adsorbed BSA-FITC on cell surface before flow cytometry measurement. Data are presented as the mean ± s.e.m. (n=6), ***$p<0.001$ analyzed by Student's t-test, one tailed.

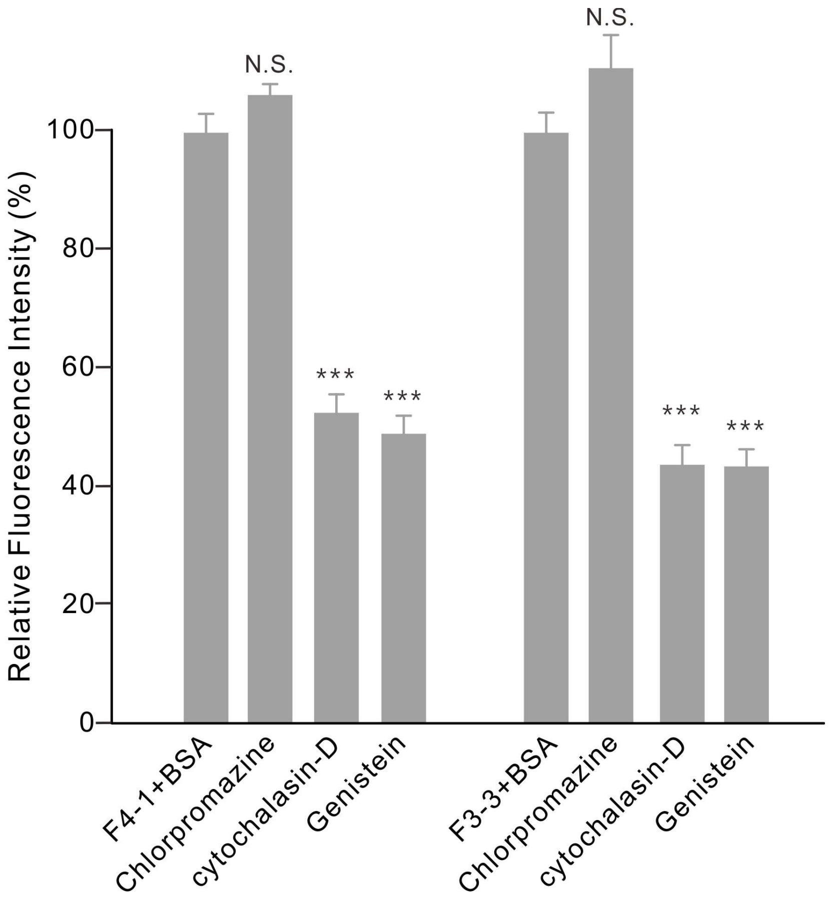


**Supplementary Fig. 3.** Internalization pathways of fluoroamphiphile/BSA complexes. The concentrations of chlorpromazine, cytochalasin-D and genistein are 20 μM, 10 μM and 700 μM, respectively. 4 μg BSA-FITC (0.3 μM) was complexed with 2 μg F4-1 and 2.5 μg F3-3, respectively. The significant inhibition of cellular uptake by cytochalasin-D and genistein suggests macropinocytosis- and caveolae-dependent endocytosis pathways. The HeLa cells were treated with 0.2 mg/mL trypan blue before flow cytometry measurement. Data are presented as the mean ± s.e.m. (n=3), $^{N.S.}p>0.05$ and $^{***}p<0.001$ analyzed by Student's t-test, one tailed.

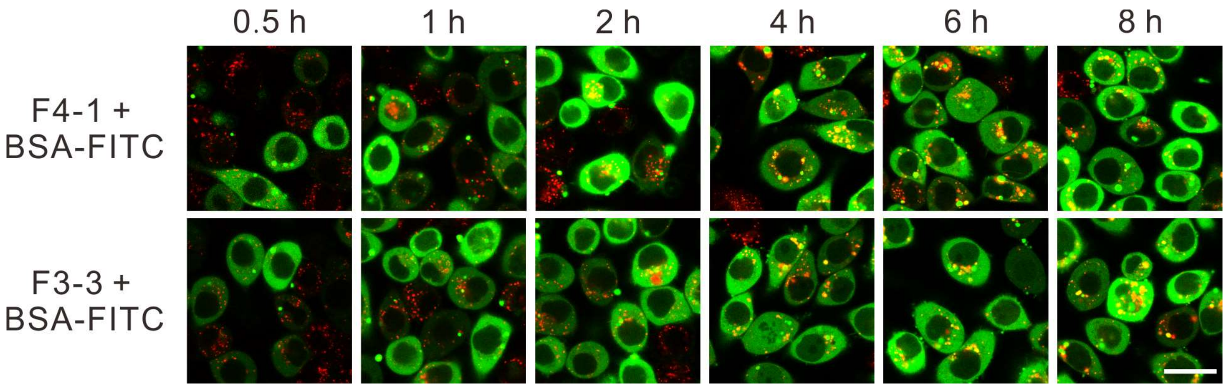


**Supplementary Fig. 4.** Images of cells treated with fluoroamphiphile/BSA complexes. HeLa cells were incubated with the complexes for 0.5 h, 1 h, 2 h, 4 h, 6 h and 8 h, respectively. 4 μg BSA-FITC (0.3 μM) was complexed with 2 μg F4-1 and 2.5 μg F3-3, respectively. The acidic organelles in HeLa cells were stained with LysoTracker Red. The scale bar is 25 μm.

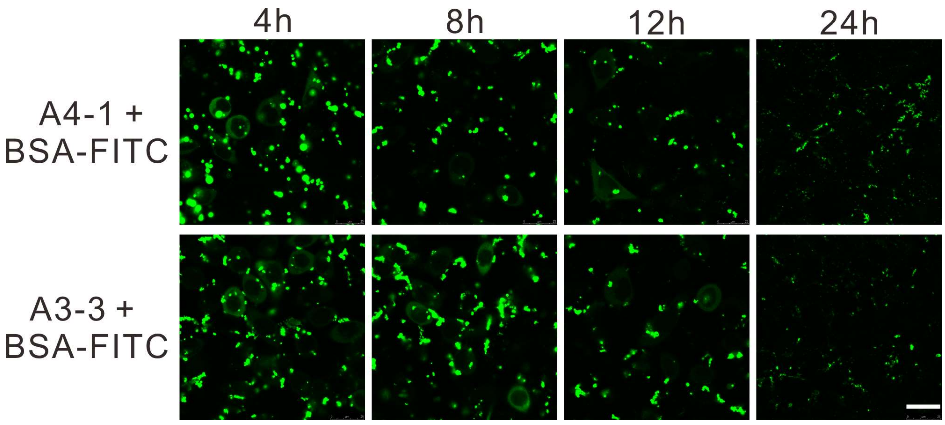


**Supplementary Fig. 5.** Images of cells treated with A4-1 or A3-3 complexes with BSA. HeLa cells were treated with the complexes for 4 h, 8 h, 12 h, and 24 h, respectively. 4 μg BSA-FITC (0.3 μM) was complexed with 1.5 μg A4-1 or A3-3. A representative result from three independent experiments. Increasing the incubation time did not show enhanced cellular uptake by the non-fluorinated control materials A4-1 and A3-3. The scale bar is 25 μm.

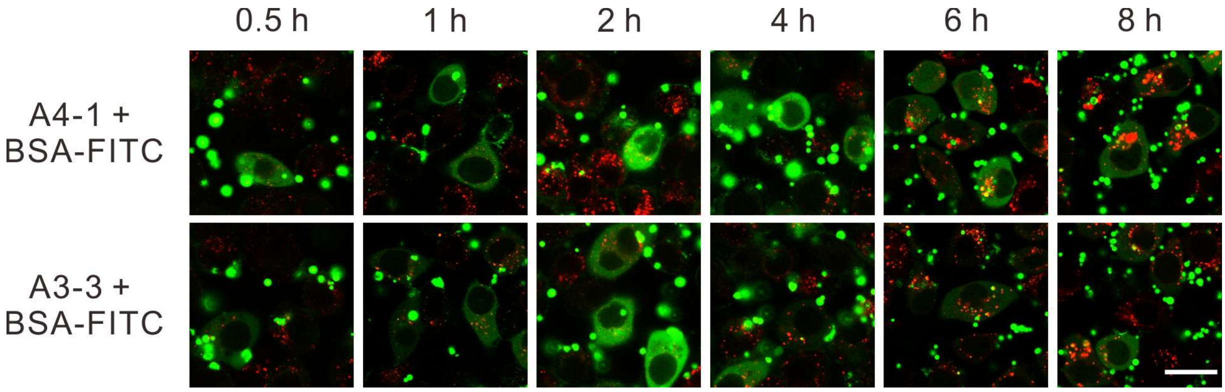


**Supplementary Fig. 6.** Images of cells treated with A4-1 or A3-3 complexes with BSA. HeLa cells were treated with the complexes for 0.5 h, 1 h, 2 h, 4 h, 6 h and 8 h, respectively. 4 µg BSA-FITC (0.3 µM) was complexed with 1.5 µg A4-1 or A3-3. The acidic organelles in HeLa cells were stained with LysoTracker Red. The scale bar is 25 µm.

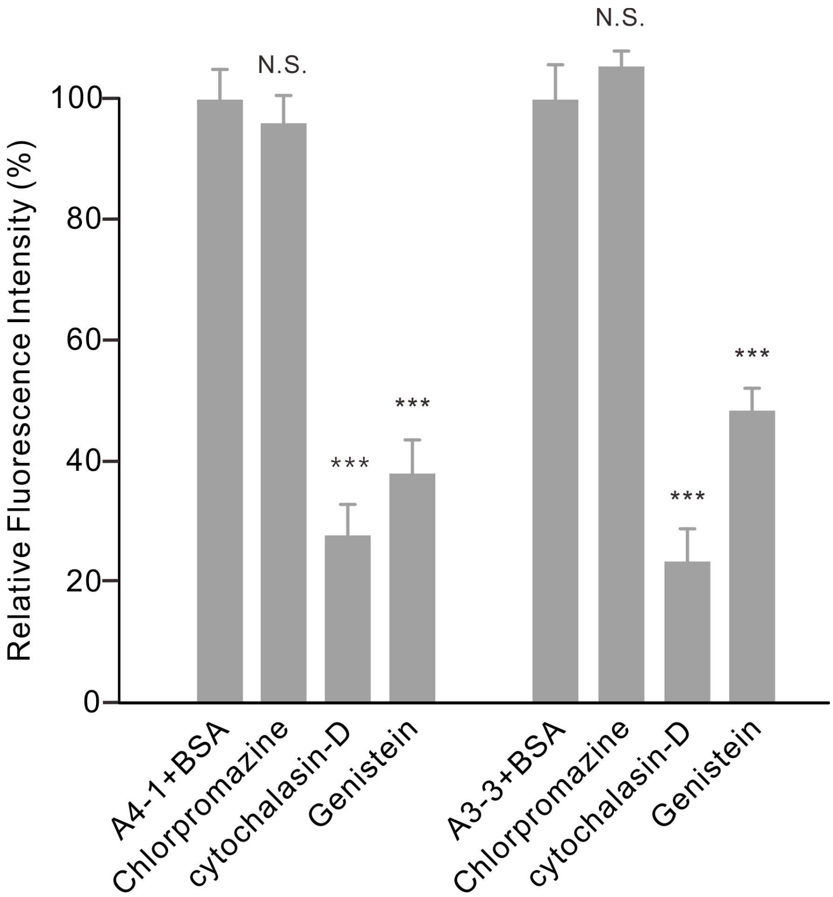


**Supplementary Fig. 7.** Internalization pathways of A4-1 and A3-3 complexes with BSA. The concentrations of chlorpromazine, cytochalasin-D and genistein are 20 μM, 10 μM and 700 μM, respectively. 4 μg BSA-FITC (0.3 μM) was complexed with 1.5 μg A4-1 or A3-3. The cells were treated with 0.2 mg/mL trypan blue before flow cytometry measurement. Data are presented as the mean ± s.e.m. (n=3), $^{N.S.}p>0.05$ and $^{***}p<0.001$ analyzed by Student's t-test, one tailed.

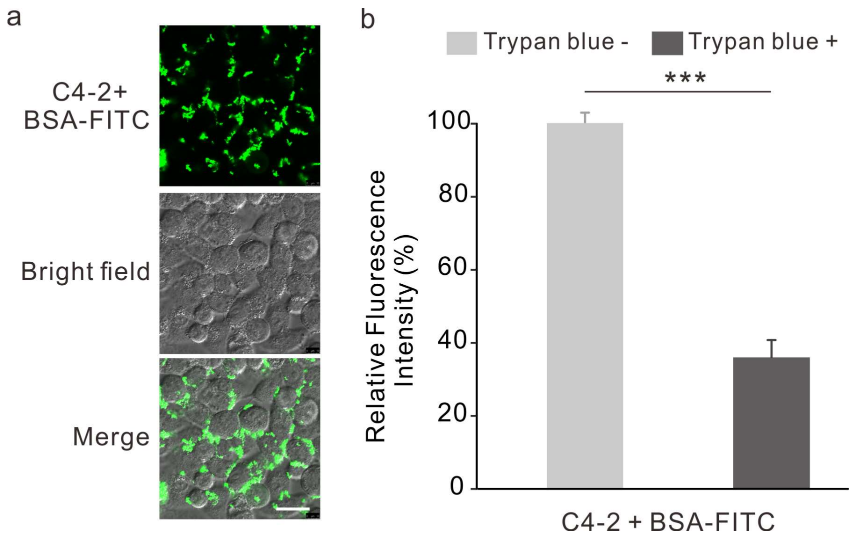


**Supplementary Fig. 8.** Cytosolic BSA-FITC delivery by C4-2. Confocal images (a) and quantitative fluorescence intensity (b) of HeLa cells treated with C4-2/BSA-FITC nanocomplexes for 4 h. 4 μg BSA-FITC (0.3 μM) was complexed with 2 μg C4-2. A representative result from three independent experiments. The scale bar is 25 μm. The trypan blue concentration is 0.2 mg/mL. Data are presented as the mean ± s.e.m. (n=3), ***$p<0.001$ analyzed by Student's t-test, one tailed.

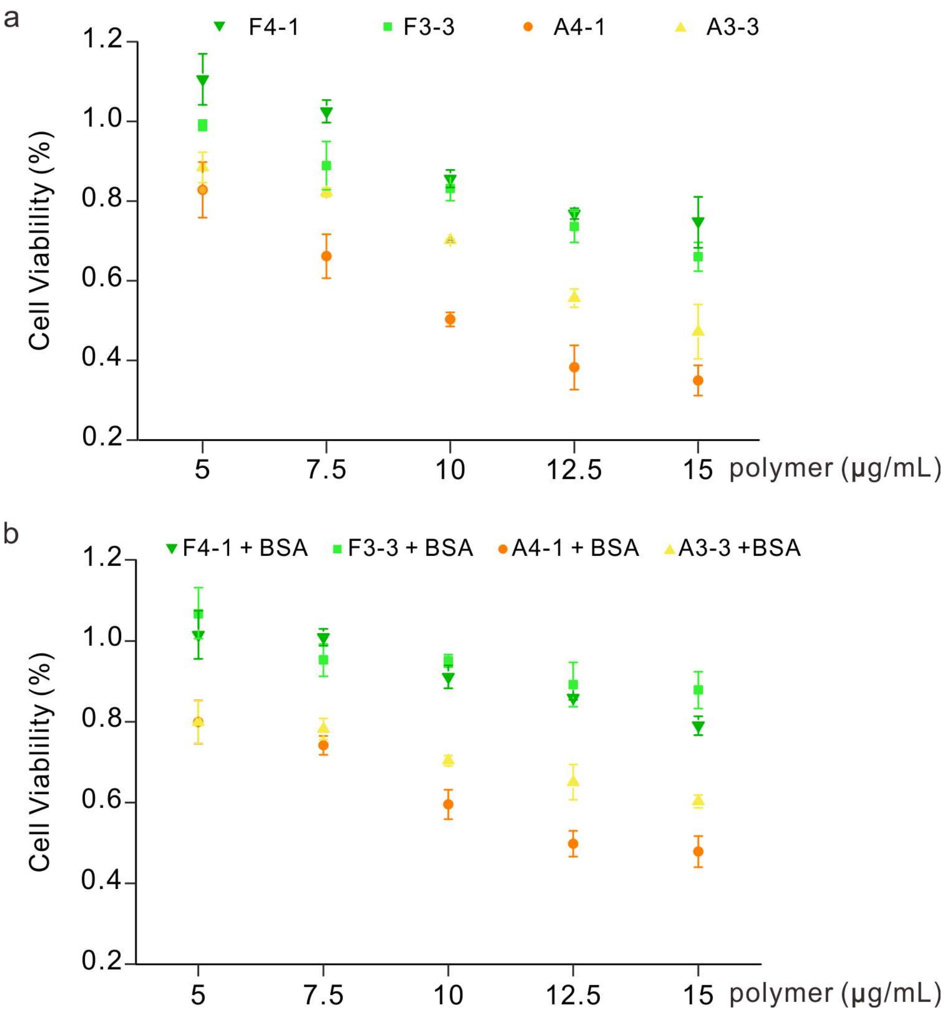


**Supplementary Fig. 9.** Cytotoxicity of the amphiphiles and related complexes. The viability of HeLa cells treated with various concentrations (5-15 μg/mL) of F4-1, F3-3, A4-1 and A3-3 alone (a) as well as their complexes with BSA (b). The BSA concentration in (b) is 20 μg/mL (0.3 μM). Data are presented as the mean ± s.e.m. (n=5).

a

| BSA+ | F1-1 | F1-2 | F1-3 | F1-4 | F2-1 | F2-2 | F2-3 | F2-4 |
|---|---|---|---|---|---|---|---|---|
| Size(nm) | 184±1.4 | 209±7.8 | 210±6 | 126±4.8 | 94±12 | 160±2 | 200±11 | 186±1. 4 |
| zeta(mV) | 48.6±0.6 | 46.3±1.3 | 45±1 | 45.9±0.6 | 24±1.7 | 44±0.8 | 46±1.2 | 46±0.4 |
| PDI | 0.18 | 0.09 | 0.06 | 0.14 | 0.22 | 0.17 | 0.16 | 0.13 |

| BSA+ | F3-1 | F3-2 | F3-3 | F3-4 | F4-1 | F4-2 | F4-3 | F4-4 |
|---|---|---|---|---|---|---|---|---|
| Size(nm) | 215±6.7 | 171.9±10 | 181±0.7 | 382±9 | 167±1.5 | 171±3.5 | 376±3.3 | 423±20 |
| zeta(mV) | 44±1 | 41.3±3 | 48.9±0.5 | 37±0.3 | 52.3±0.5 | 52±5.4 | 35±0.2 | 39±0.5 |
| PDI | 0.09 | 0.13 | 0.15 | 0.2 | 0.07 | 0.1 | 0.25 | 0.26 |

b

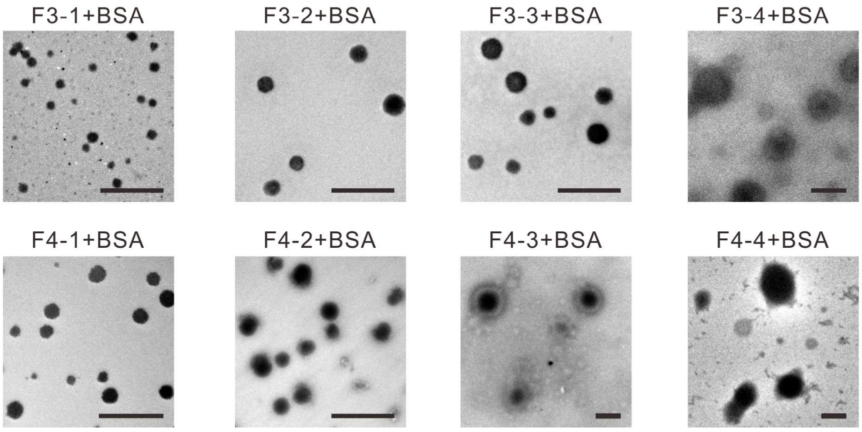


**Supplementary Fig. 10.** Characterization of fluoroamphiphile/BSA complexes. Size, zeta-potential, PDI (a) and TEM images (b) of the complexes. The molar ratio of fluoroamphiphiles to BSA is 1: 1. Scale bars: 500 nm. Data are presented as the mean ± s.d. (n=3).

| BSA- | F3-4 | F4-3 | F4-4 |
|---|---|---|---|
| Size(nm) | 294±14 | 278±11 | 392±49 |
| Zeta(mV) | 54±0.2 | 56±0.5 | 56±0.3 |
| PDI | 0.22 | 0.17 | 0.25 |

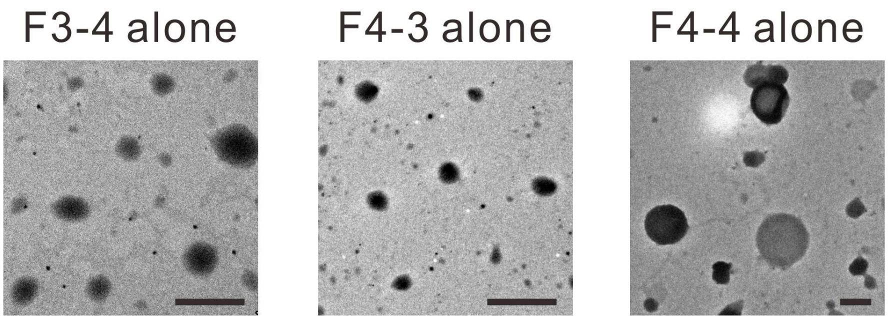


**Supplementary Fig. 11.** Characterization of the fluoroamphiphile F3-4, F4-3 and F4-4 alone. The size, zeta-potential, PDI and TEM images of F3-4, F4-3 and F4-4 alone were shown. Scale bars: 500 nm. Data are presented as the mean ± s.d. (n=3).

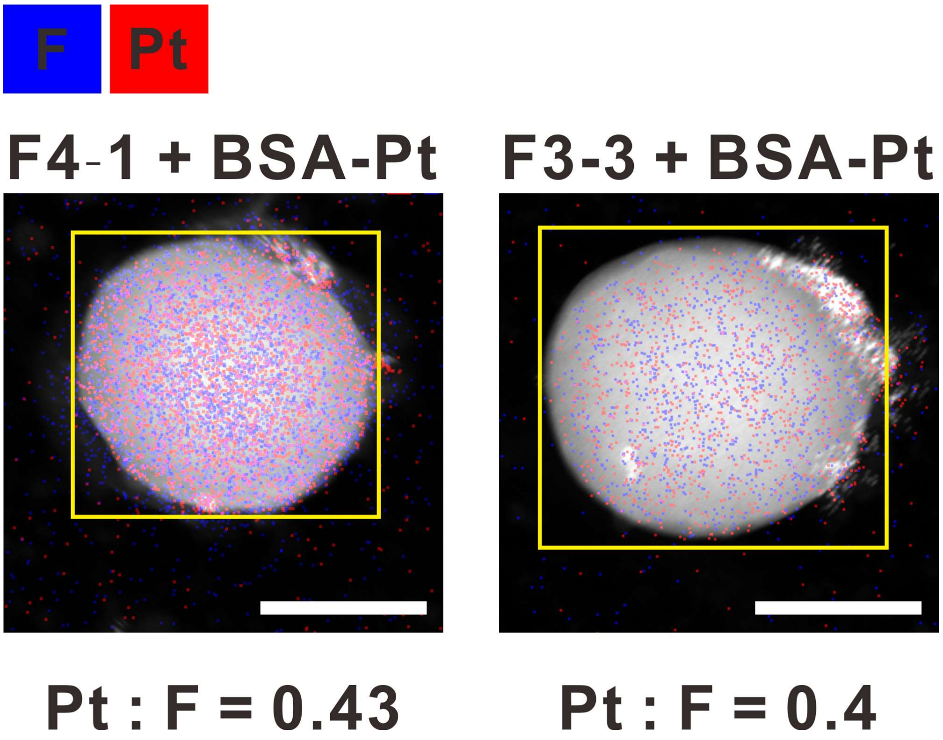


**Supplementary Fig. 12.** Element mappings of F4-1 and F3-3 complexes with BSA-Pt. Pt: F means the molar ratio of Pt and F elements within the square frame. Scale bars: 100 nm.

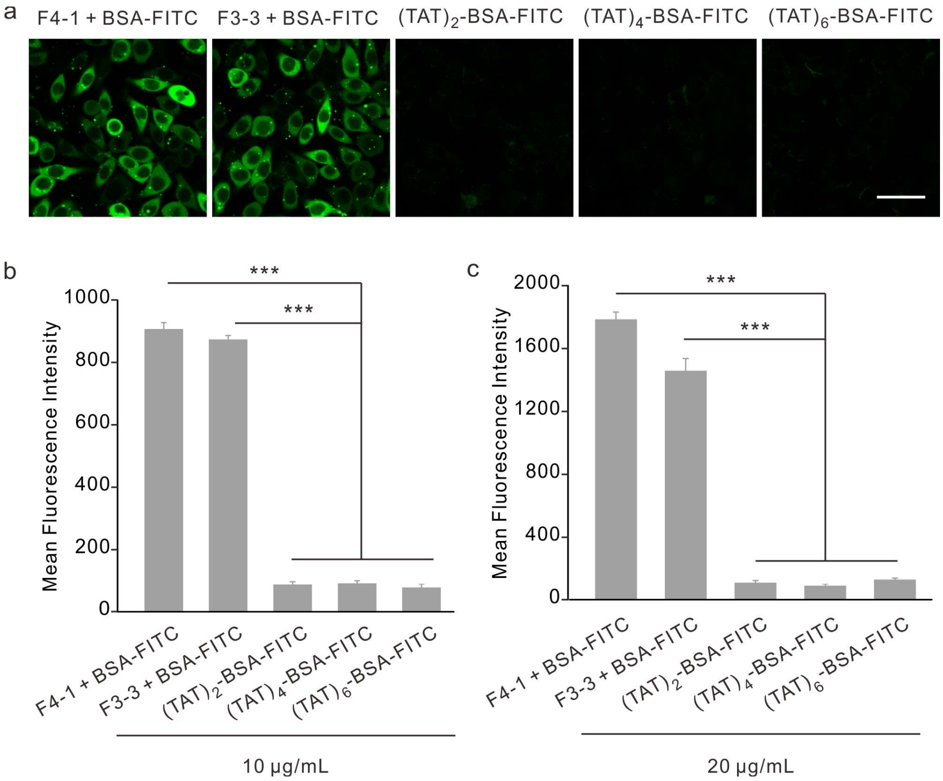


**Supplementary Fig. 13.** Fluoroamphiphiles *versus* TAT conjugates in protein delivery. (a) Confocal images of HeLa cells treated with F4-1/BSA-FITC, F3-3/BSA-FITC and TAT-conjugated BSA-FITC for 4 h. TAT peptide with an N-terminal cysteine was conjugated to BSA-FITC using a SMCC linker. The BSA-FITC was conjugated with 2, 4, or 6 TAT chains, respectively. The BSA concentration is 20 μg/mL. The scale bar is 50 μm. (b, c) Fluorescence intensity of HeLa cells treated with F4-1/BSA-FITC, F3-3/BSA-FITC and TAT-conjugated BSA-FITC for 4 h at BSA concentrations of 10 μg/mL (0.15 μM, b) and 20 μg/mL (0.3 μM, c), respectively analyzed by flow cytometry. Data are presented as the mean ± s.e.m. (n=3). ***$p<0.001$ analyzed by Student's t-test, one tailed.

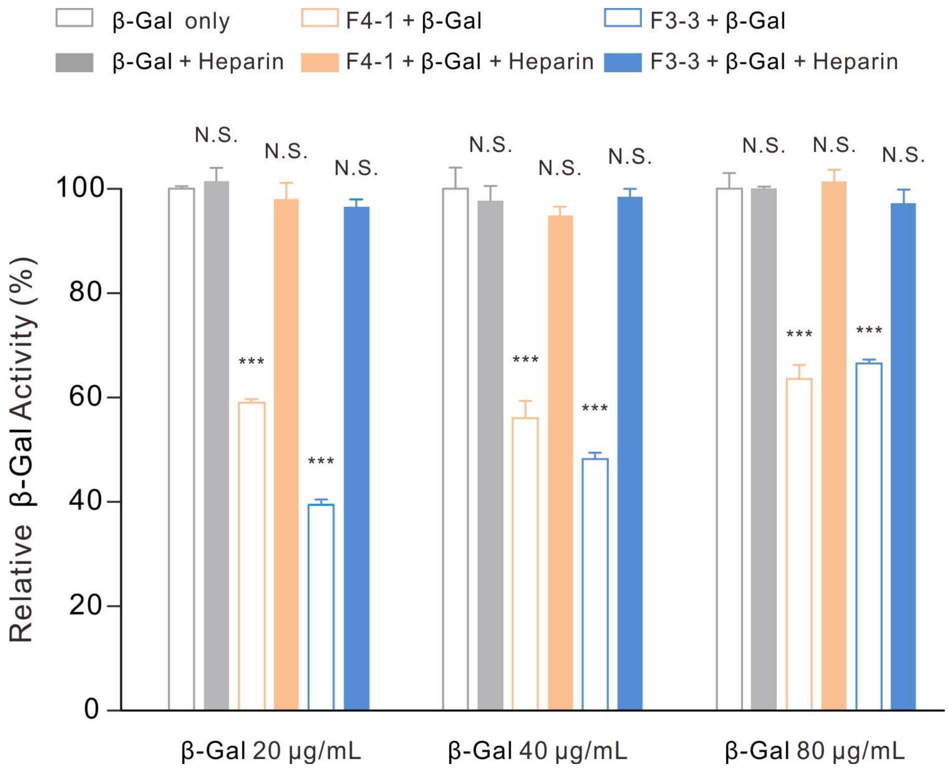


**Supplementary Fig. 14.** Relative β-Gal activity determined by X-gal staining. The recovery of β-Gal activity was conducted by the addition of 0.1 mg/mL heparin sodium into the complex solutions. The concentration of F4-1 and F3-3 is fixed at 40 μg/mL. Data are presented as the mean ± s.e.m. (n=3). $^{N.S.}p>0.05$ and $^{***}p<0.001$ analyzed by Student's t-test, one tailed.

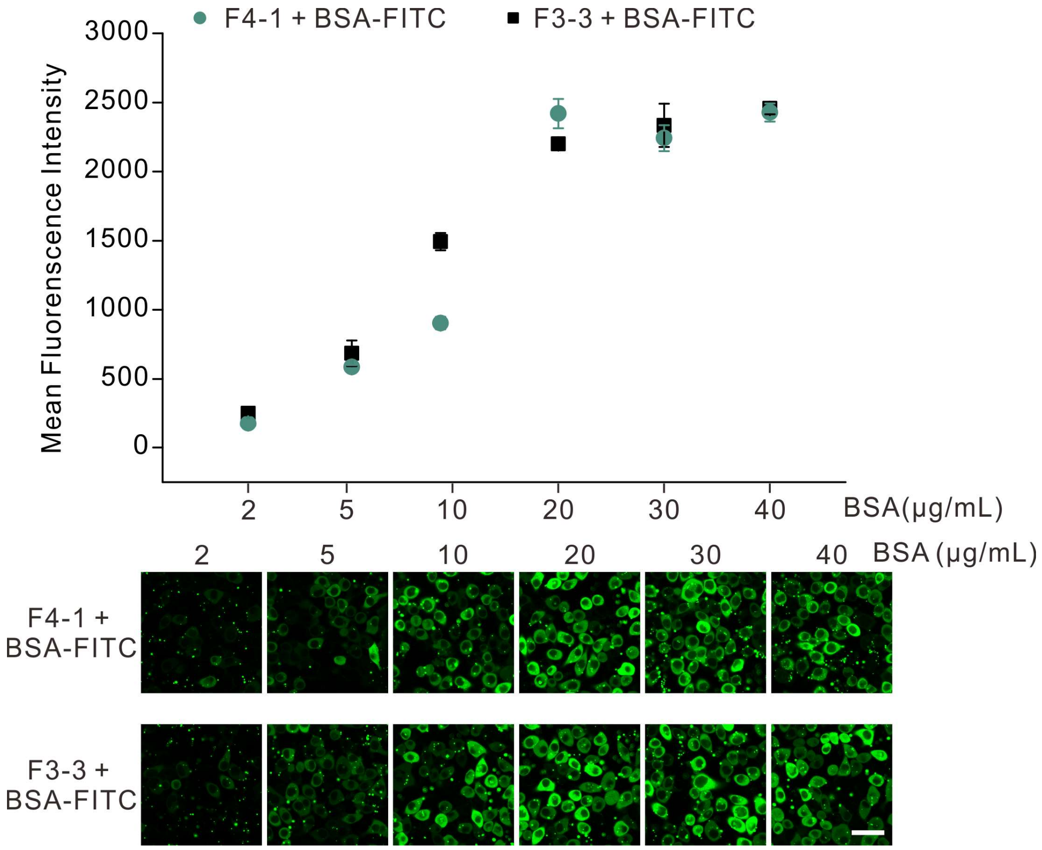


**Supplementary Fig. 15.** The effect of BSA dose in cytosolic protein delivery. (a) Fluorescence intensity of HeLa cells treated with F4-1/BSA-FITC or F3-3/BSA-FITC complexes for 4 h at different protein concentrations analyzed by flow cytometry. Data are presented as the mean ± s.e.m. (n=3). (b) Confocal images of HeLa cells treated with F4-1/BSA-FITC or F3-3/BSA-FITC complexes for 4 h. The polymer concentrations for F4-1 and F3-3 are 10 and 12.5 μg/mL, respectively. The scale bar is 50 μm.

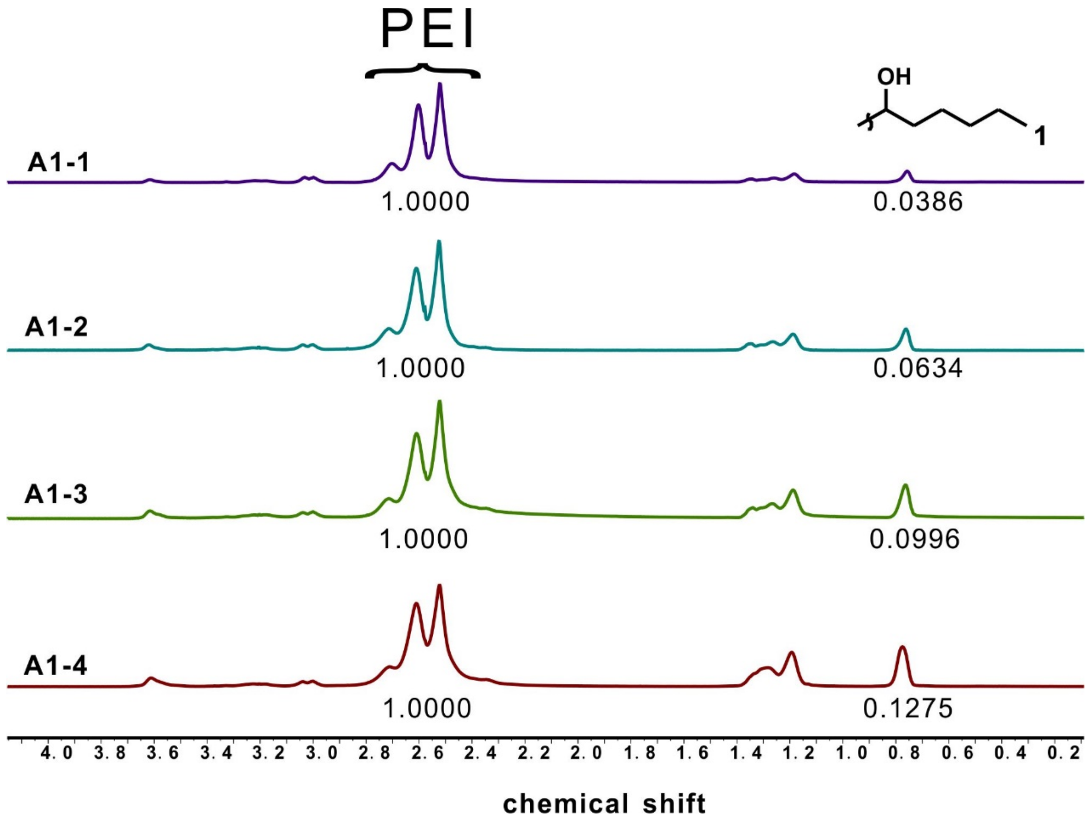


**Supplementary Fig. 16.** $^{1}H$ NMR spectra of A1-modified PEIs.

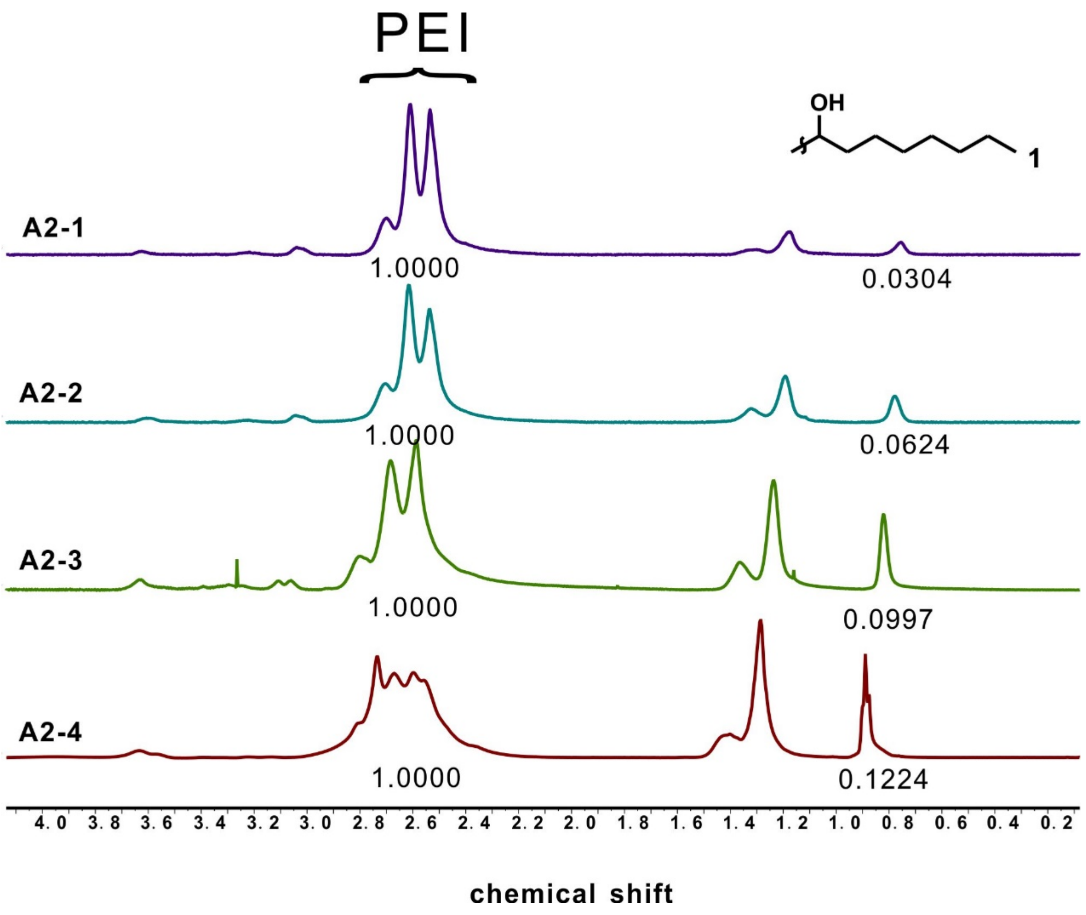


**Supplementary Fig. 17.** $^1$H NMR spectra of A2-modified PEIs.

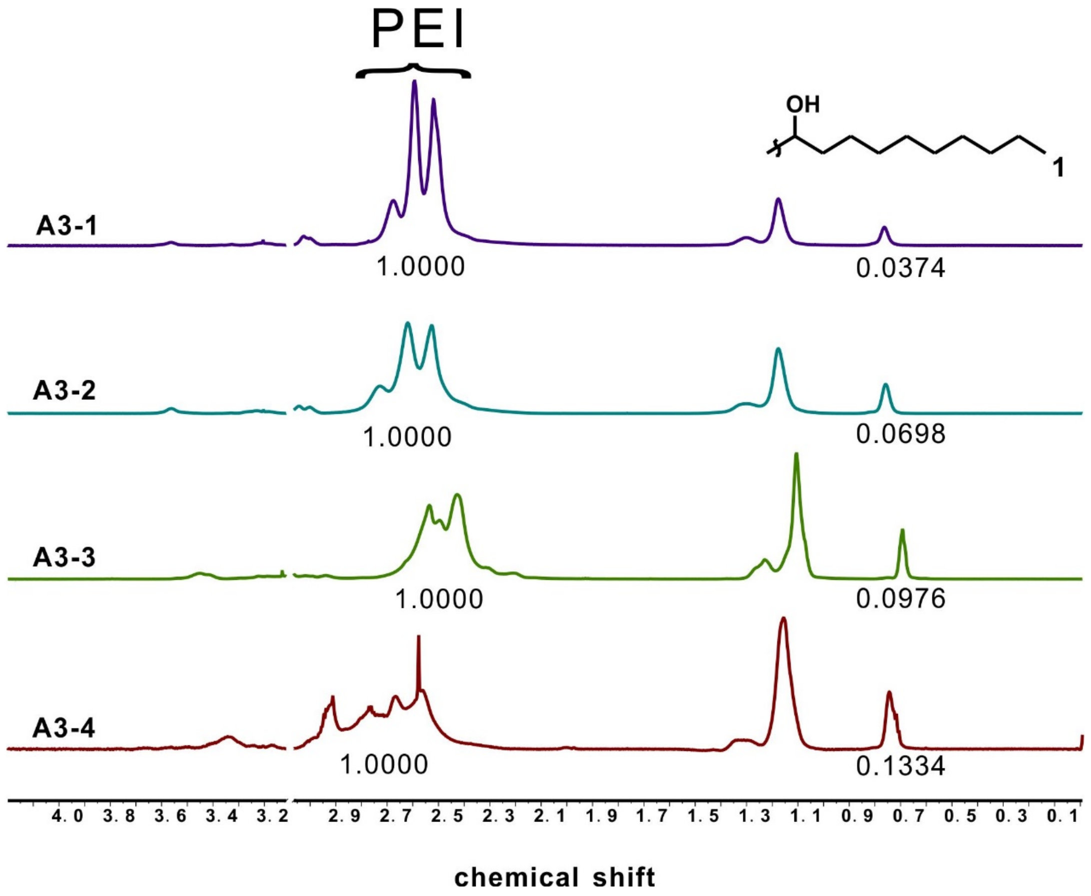


**Supplementary Fig. 18.** $^{1}$H NMR spectra of A3-modified PEIs.

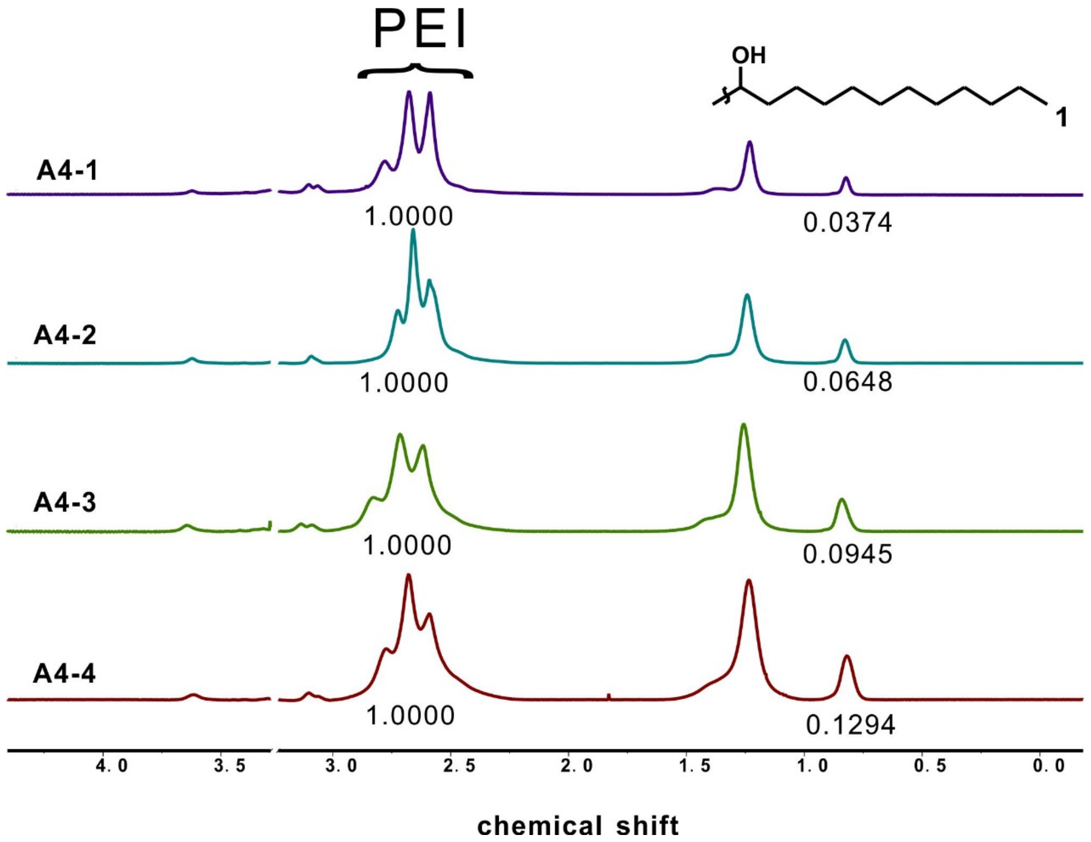


**Supplementary Fig. 19.** $^{1}$H NMR spectra of A4-modified PEIs.

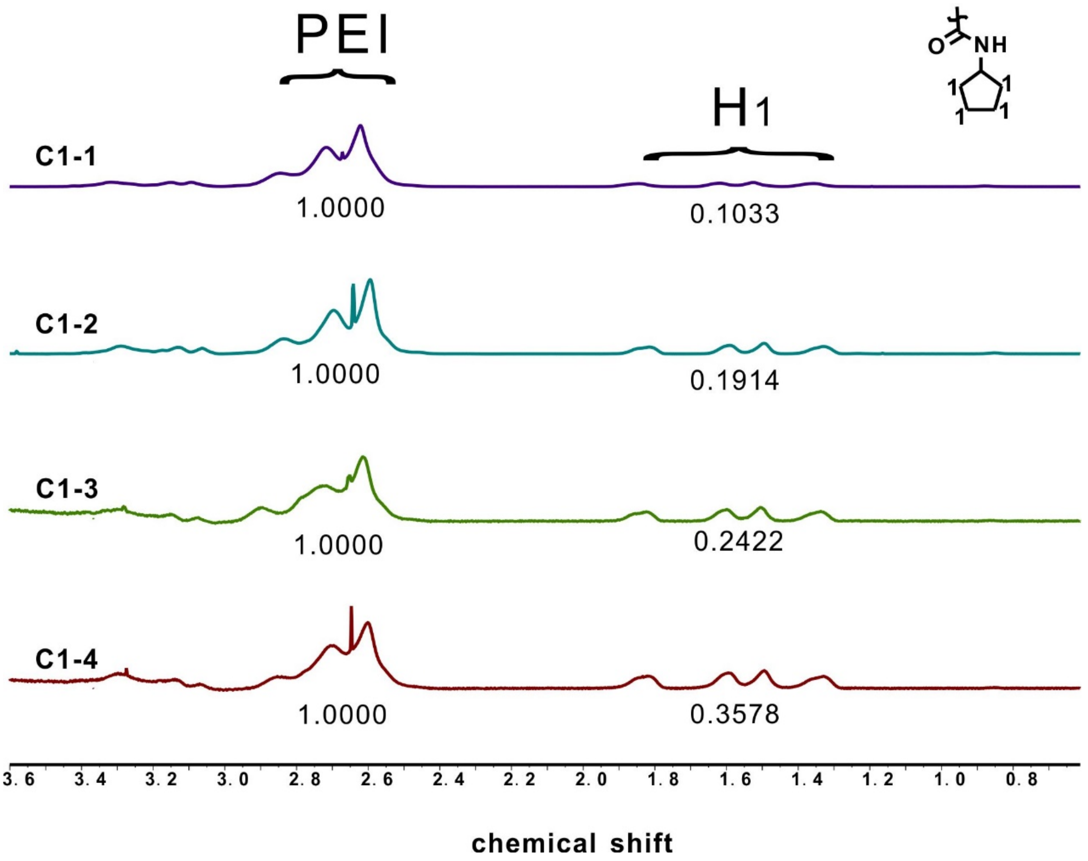


**Supplementary Fig. 20.** $^1$H NMR spectra of C1-modified PEIs.

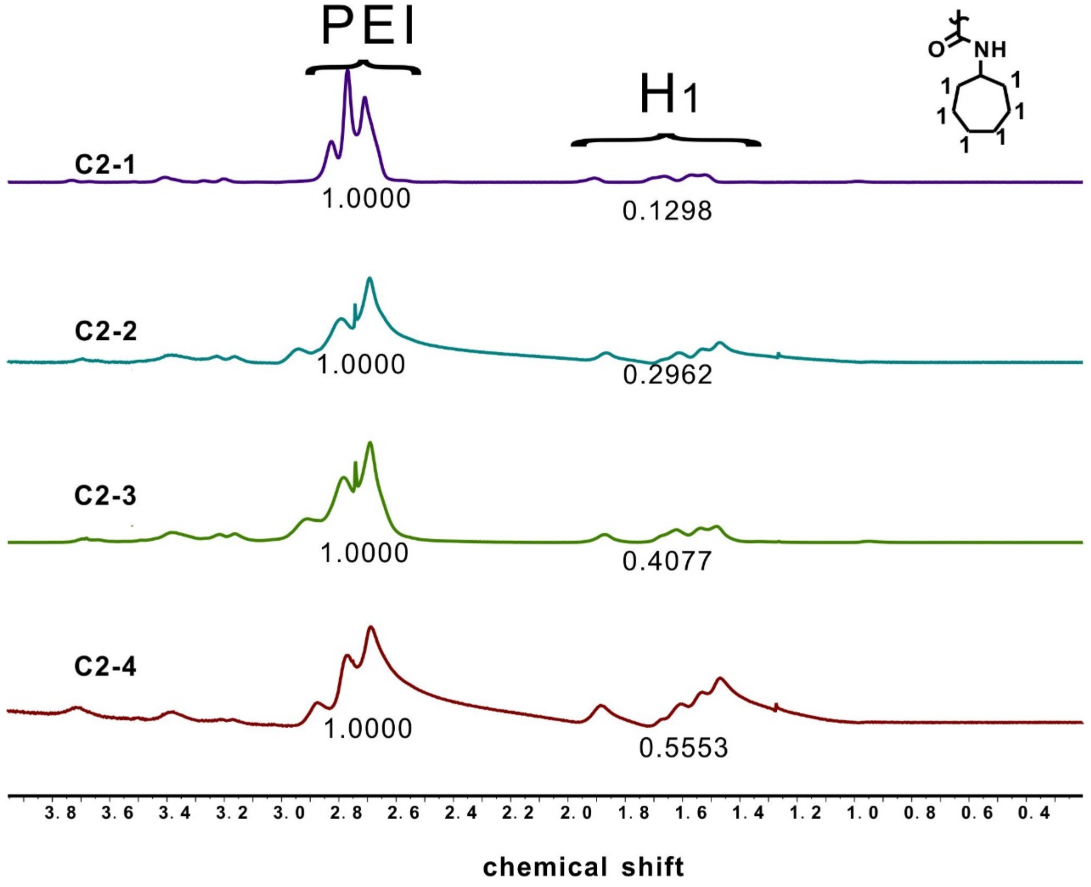


**Supplementary Fig. 21.** $^{1}$H NMR spectra of C2-modified PEIs.

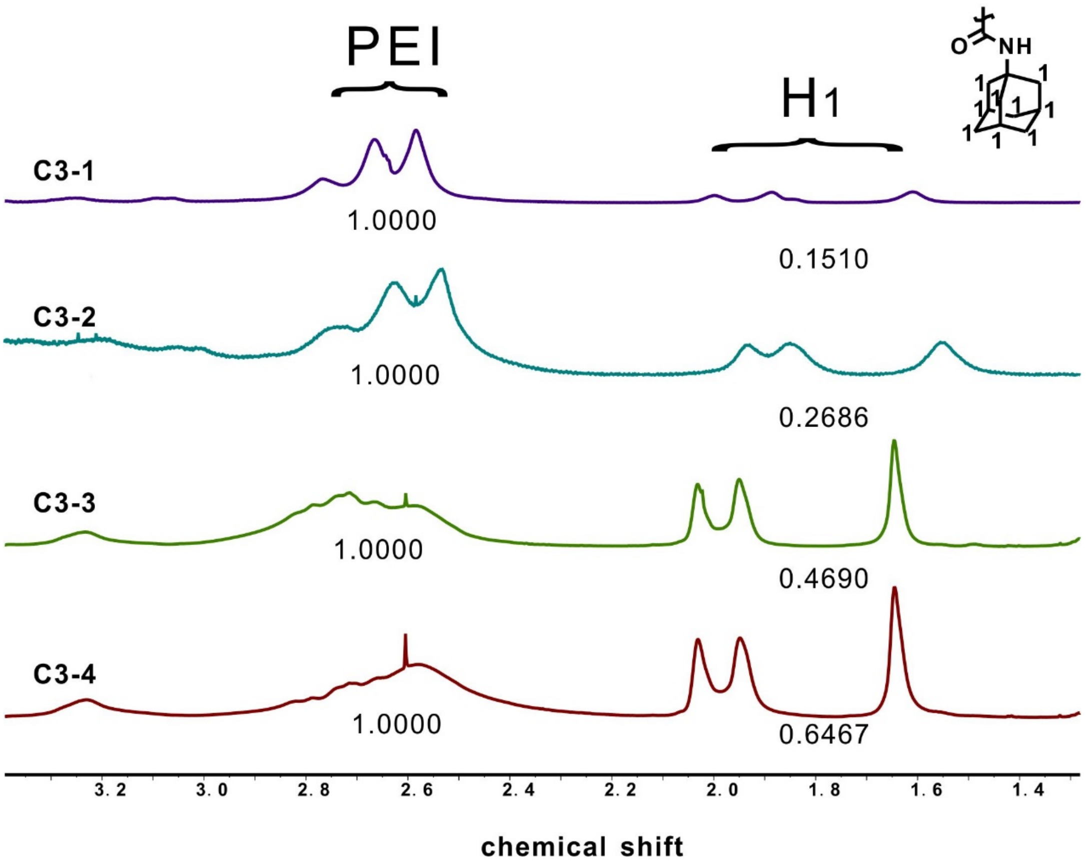


**Supplementary Fig. 22.** $^{1}$H NMR spectra of C3-modified PEIs.

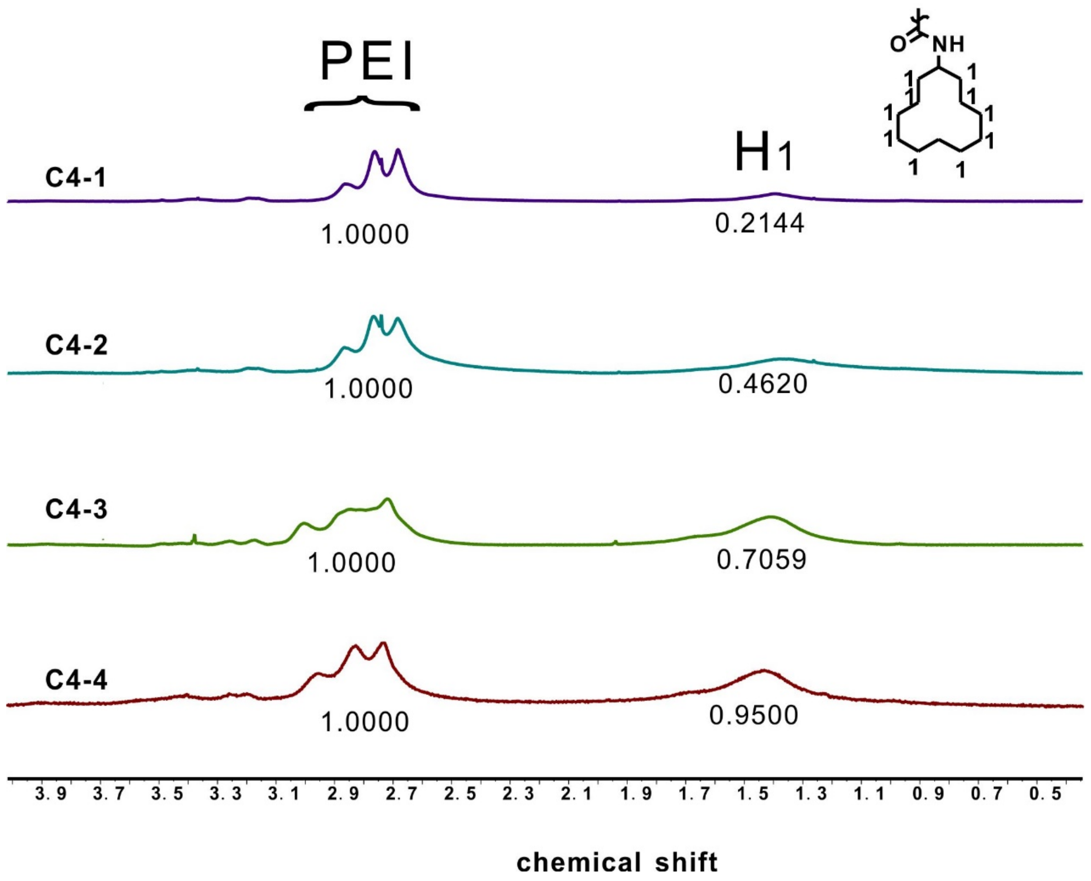


**Supplementary Fig. 23.** $^1$H NMR spectra of C4-modified PEIs.

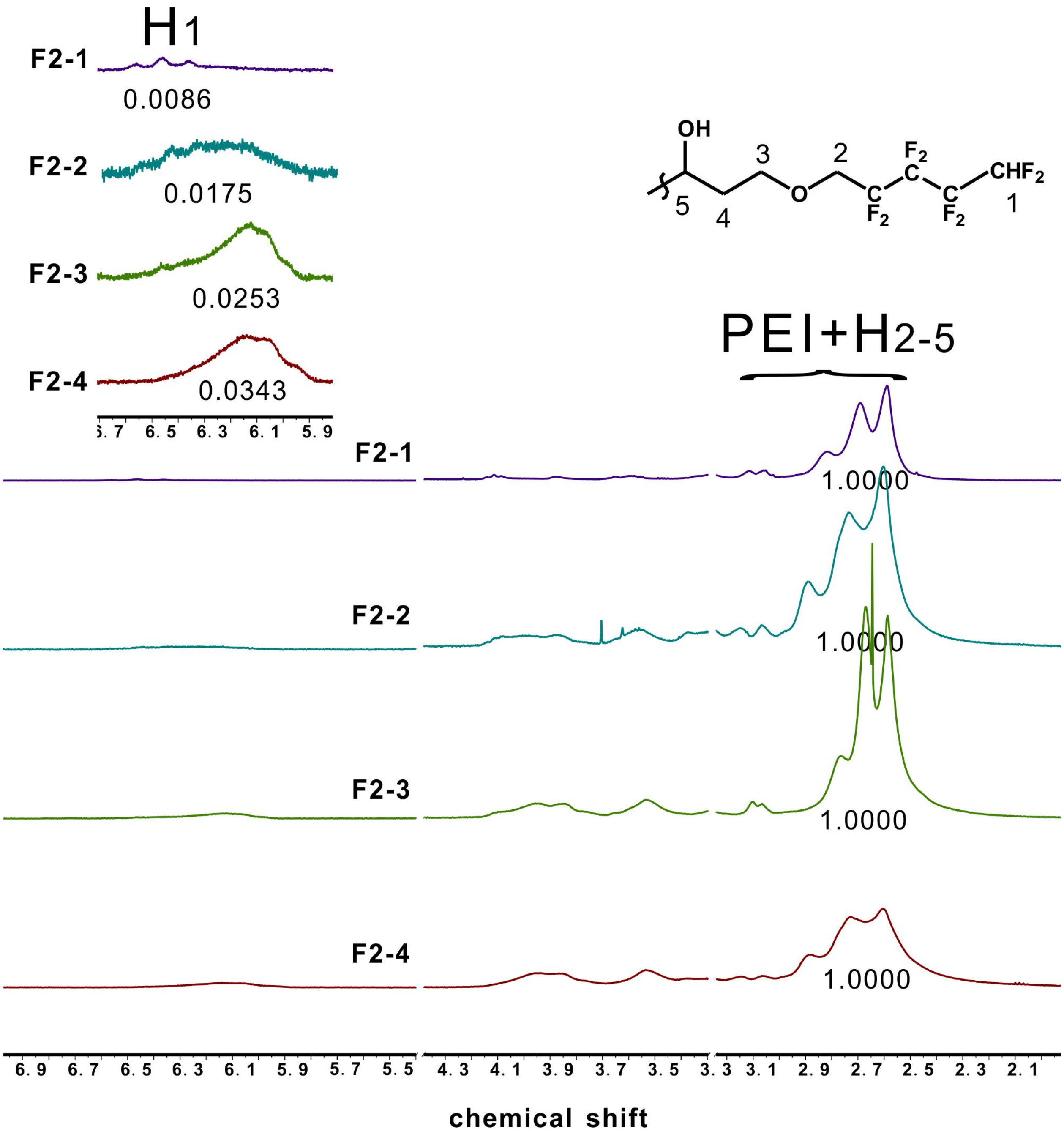


**Supplementary Fig. 24.** $^1$H NMR spectra of F2-modified PEIs.

# Supplementary Tables

**Supplementary Table 1**. Synthesis, characterization and screening results of amphiphiles in the library.

| Ligand | Chemical | Feeding ratio[a] | Conjugated number[b] | Fluorine content (wt%)[c] | Optimal dose[d] | Mean fluorescence before quench[e] | Mean fluorescence after quench[f] | Product name[g] |
|---|---|---|---|---|---|---|---|---|
| A1 | 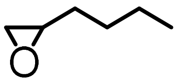 | 36 | 30 | - | 1 μg | 32.28 | 24.66 | A1-1[h] |
| | | 72 | 49 | - | 1 μg | 33.62 | 21.67 | A1-2[h] |
| | | 108 | 77 | - | 1 μg | 49.10 | 27.72 | A1-3[h] |
| | | 143 | 96 | - | 1 μg | 48.43 | 25.16 | A1-4[h] |
| A2 | 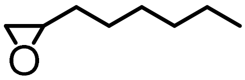 | 72 | 24 | - | 0.5 μg | 35.77 | 33.56 | A2-1[h] |
| | | 143 | 48 | - | 1 μg | 358.51 | 239.69 | A2-2[h] |
| | | 172 | 77 | - | 1.5 μg | 562.41 | 346.53 | A2-3[h] |
| | | 286 | 95 | - | 1.5 μg | 239.01 | 186.56 | A2-4[h] |
| A3 | 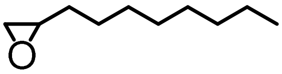 | 36 | 29 | - | 1 μg | 687.36 | 523.17 | A3-1[h] |
| | | 72 | 54 | - | 1 μg | 1018.80 | 501.33 | A3-2[h] |
| | | 86 | 76 | - | 1.5 μg | 1333.91 | 360.16 | A3-3[h] |
| | | 108 | 101 | - | 1.5 μg | 826.09 | 284.89 | A3-4[h] |
| A4 | 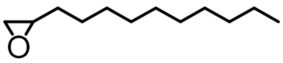 | 36 | 29 | - | 1.5 μg | 1099.52 | 539.71 | A4-1[h] |
| | | 72 | 50 | - | 1.5 μg | 1090.27 | 516.94 | A4-2[h] |
| | | 143 | 73 | - | 1.5 μg | 1229.75 | 172.53 | A4-3[h] |
| | | 172 | 92 | - | 1.5 μg | 1345.97 | 124 | A4-4[h] |
| C1 | 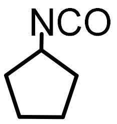 | 36 | 30 | - | 1 μg | 36.06 | 10.82 | C1-1[h] |
| | | 72 | 56 | - | 1 μg | 36.33 | 10.90 | C1-2[h] |
| | | 90 | 73 | - | 1 μg | 39.99 | 16.00 | C1-3[h] |
| | | 125 | 104 | - | 1 μg | 43.57 | 13.61 | C1-4[h] |
| C2 | 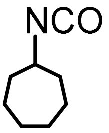 | 25 | 25 | - | 1 μg | 43.07 | 16.48 | C2-1[h] |
| | | 50 | 50 | - | 1 μg | 78.05 | 33.5 | C2-2[h] |

| | | | | | | | | |
|---|---|---|---|---|---|---|---|---|
| | | 75 | 75 | - | 1 μg | 141.07 | 89.66 | C2-3 [h] |
| | | 100 | 92 | - | 1 μg | 168.23 | 93.47 | C2-4 [h] |
| C3 | 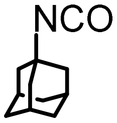 | 25 | 25 | - | 1 μg | 73.25 | 27.98 | C3-1 [h] |
| | | 50 | 45 | - | 1 μg | 145.63 | 53.49 | C3-2 [h] |
| | | 75 | 78 | - | 1 μg | 446.56 | 120.90 | C3-3 [h] |
| | | 100 | 104 | - | 1 μg | 197.91 | 66.75 | C3-4 [h] |
| C4 | 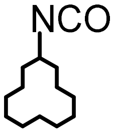 | 25 | 23 | - | 2 μg | 160.2 | 79.37 | C4-1 [h] |
| | | 50 | 54 | - | 2 μg | 493.43 | 168.61 | C4-2 [h] |
| | | 75 | 73 | - | 2 μg | 187.95 | 92.52 | C4-3 [h] |
| | | 100 | 100 | - | 2 μg | 194.11 | 129 | C4-4 [h] |
| F1 | 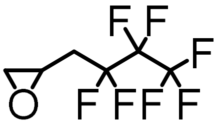 | 72 | 27 | 12.02 | 1 μg | 65.46 | 37.34 | F1-1 [i] |
| | | 108 | 52 | 18.82 | 1 μg | 180.29 | 147.56 | F1-2 [i] |
| | | 143 | 68 | 22.32 | 1 μg | 330.25 | 301.85 | F1-3 [i] |
| | | 215 | 98 | 27.62 | 2 μg | 520.47 | 469.51 | F1-4 [i] |
| F2 | 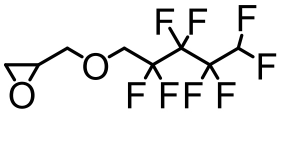 | 25 | 21 | 10.25 | 1 μg | 62.76 | 57.65 | F2-1 [h] |
| | | 50 | 46 | 18.21 | 1 μg | 86.02 | 89.68 | F2-2 [h] |
| | | 72 | 78 | 24.86 | 1 μg | 359.84 | 389.94 | F2-3 [h] |
| | | 108 | 105 | 28.73 | 2 μg | 685.04 | 627.23 | F2-4 [h] |
| F3 | 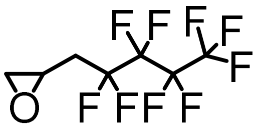 | 36 | 28 | 14.69 | 1 μg | 201.54 | 190.64 | F3-1 [i] |
| | | 72 | 54 | 23.13 | 2 μg | 906.44 | 846.97 | F3-2 [i] |
| | | 108 | 76 | 28.27 | 2.5 μg | 1439.07 | 1282.80 | F3-3 [i] |
| | | 143 | 102 | 32.81 | 2.5 μg | 47.92 | 53.69 | F3-4 [i] |
| F4 | 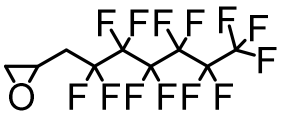 | 36 | 30 | 20.49 | 2 μg | 1712.29 | 1589.31 | F4-1 [i] |

| | | | | | | | | |
|---|---|---|---|---|---|---|---|---|
| | | 72 | 51 | 28.45 | 2 μg | 1021.94 | 956.18 | F4-2[i] |
| | | 108 | 76 | 30.07 | 2.5 μg | 223.97 | 216.38 | F4-3[i] |
| | | 143 | 112 | 41.52 | 2.5 μg | 33.13 | 32.55 | F4-4[i] |

**Notes:**

[a]Feeding ratio: the feeding molar ratio of chemical to PEI during the synthesis of amphiphiles.

[b]Conjugated number: the average number of ligands conjugated on each PEI according to $^{1}$H NMR or fluorine element analysis.

[c]Fluorine content (wt%): the weight percent of fluorine in the synthesized fluoroamphiphiles.

[d]Optimal dose: For each material, the dose is chosen at which the highest fluorescence intensity of cells is achieved and the complex is not cytotoxic on the transfected cells. During the screening experiments, 4 μg BSA-FITC was mixed with 0.5, 1, 1.5, 2, and 2.5 μg amphiphiles, respectively.

[e]Mean fluorescence before quench: the fluorescence intensity of HeLa cells transfected by amphiphile/BSA-FITC complexes at optimal doses.

[f]Mean fluorescence after quench: The transfected cells at optimal dose for each material were also incubated with trypan blue to quench the BSA-FITC physically adsorbed on cell membrane, and then the fluorescence intensity of cells were quantitatively analyzed by flow cytometry.

[g]Product name: The synthesized products were termed according to the hydrophobic substitutes and the feeding ratio. [h]means the products are characterized by $^{1}$H NMR; [i]means the products are characterized by fluorine element analysis (CAS Shanghai Institute of Organic Chemistry, China).

**Supplementary Table 2**. Size and PDI values of polymer/protein complexes at different protein to polymer weight ratios. The polymer concentration is 0.55 μM. Data are presented as mean±s.d. (n=3).

| BSA : F4-1 | 2 | 1.5 | 1 | 0.5 | 0.2 | 0.1 |
|---|---|---|---|---|---|---|
| Size (nm) | 284 ± 10.7 | 213 ± 2.7 | 190 ± 4.3 | 227 ± 2.2 | 238 ± 4.4 | 265 ± 5.1 |
| PDI | 0.02 | 0.16 | 0.19 | 0.25 | 0.12 | 0.26 |

| BSA : F3-3 | 1 | 0.75 | 0.5 | 0.2 | 0.15 | 0.1 |
|---|---|---|---|---|---|---|
| Size (nm) | 166 ± 3.9 | 189 ± 7 | 209 ± 5.4 | 236 ± 9 | 250± 12 | 282 ± 13 |
| PDI | 0.17 | 0.19 | 0.18 | 0.23 | 0.24 | 0.3 |

| β-Gal : F4-1 | 2 | 1.5 | 1 | 0.5 | 0.2 | 0.1 |
|---|---|---|---|---|---|---|
| Size (nm) | 251 ± 7.1 | 234 ± 8 | 275 ± 49 | 252 ± 17 | 222 ± 6.5 | 222 ± 3.2 |
| PDI | 0.13 | 0.11 | 0.23 | 0.18 | 0.2 | 0.14 |

| β-Gal : F3-3 | 1.5 | 1 | 0.5 | 0.2 | 0.15 | 0.1 |
|---|---|---|---|---|---|---|
| Size (nm) | 239 ± 7.1 | 266 ± 3.9 | 230 ± 18 | 236 ± 14 | 238 ± 8 | 256 ± 13 |
| PDI | 0.08 | 0.2 | 0.22 | 0.23 | 0.23 | 0.3 |

| Saporin : F4-1 | 3 | 2.5 | 2 | 1.5 | 1 | 0.5 |
|---|---|---|---|---|---|---|
| Size (nm) | 262 ± 8 | 176± 1 | 146 ± 4.3 | 103 ± 2.4 | 112 ± 2.8 | 152 ± 7 |
| PDI | 0.11 | 0.2 | 0.18 | 0.3 | 0.23 | 0.27 |

| Saporin : F3-3 | 3 | 2.5 | 2 | 1.5 | 1 | 0.5 |
|---|---|---|---|---|---|---|
| Size (nm) | 272 ± 8.5 | 173 ± 0.2 | 187 ± 3.5 | 165 ± 7.5 | 173 ± 1.8 | 225 ± 7.4 |
| PDI | 0.05 | 0.1 | 0.09 | 0.16 | 0.26 | 0.3 |

## Supplementary Methods

**Materials.** Branched PEI with a molecular weight of 25 kDa, cyclopentyl isocyanate, cycloheptyl isocyanate, 1-adamantyl isocyanate, cyclododecyl isocyanate, 1,2-epoxydodecane, 3-(1H,1H,5H-perfluoropentyloxy)-1,2-epoxypropane, FITC and saporin from *Saponaria officinalis* seeds were obtained from Sigma-Aldrich (St. Louis, MO). β-Gal, 1,2-epoxyhexane, 1,2-epoxydecane, 3-(perfluoropropyl)-1,2-propenoxide, 3-(perfluorobut-1-yl)-1,2-propenoxide, and 3-(perfluorohex-1-yl)-1,2-propenoxide were obtained from J&K Scientific (Shanghai, China). 1,2-Epoxyoctane and BSA were purchased from Aladdin (Shanghai, China). 3-(4, 5-Dimethylthiazol-2-yl)-2,5-diphenyltetrazolium bromide (MTT) was purchased from Sangon Biotech (Shanghai, China). Succinimidyl 4-(N-maleimidomethyl)cyclohexane-1-carboxylate (SMCC) was purchased from Macklin (Shanghai, China). CGRKKRRQRRR (TAT) was synthesized by GL Biochem. (Shanghai, China), and FITC-conjugated GRKKRRQRRREKIKRPRSSNAETL by Shanghai Top-peptide Biotechnol. (Shanghai, China). PULSin™ was obtained from Polyplus Transfection (France). BCA assay kit, β-Gal staining kit and β-Gal assay kit were purchased from Beyotime (Jiangsu, China). Trypan blue was purchased from Yesen (Shanghai, China).

**Synthesis of modified PEIs.** Functionalized PEIs were synthesized by dropwise addition of isocyanates bearing cycloalkanes or epoxides bearing alkanes/fluoroalkanes into bPEI in methanol at different molar ratios (Supplementary Table 1). Generally, 30 mg PEI was used for each sample. The solutions were stirred at room temperature for 48 h. The products were purified by intensive dialysis against methanol and double-distilled water (MWCO 3500 Da), and lyophilized. The synthesized materials were characterized by $^{1}H$ NMR (Varian 699.804 MHz) in $D_2O$ (Supplementary Figs. 16-24) or fluorine element analysis to calculate the average number of conjugated substituents.

**Synthesis of BSA-FITC.** BSA (10 mg/mL, 0.15 mM) was dissolved in phosphate buffer saline (PBS, pH 7.4) and the solution was added with FITC dissolved in DMSO at a BSA/FITC molecular ratio of 1:3. The mixture was stirred at room temperature for 24 h and intensively dialyzed against PBS and distilled water (MWCO 3500 Da). The product was lyophilized to obtain BSA-FITC as yellow powders, and stored at -20 °C for further use.

**Synthesis of BSA-Pt.** 0.1 mg BSA was dispersed in 10 mL PBS, and 0.5 mL $H_2PtCl_6$ (38.6 mM) in PBS was added into the above solution. The mixture was magnetically stirred at dark for 30 min. After that, 0.25 mL $NaBH_4$ (0.5 M) was added in the reaction solution to reduce the platinum (IV) ions. After stirring for 2 h, the solution was transferred to a dialysis bag (MWCO 3500 Da), and then intensively dialyzed against deionized water.

**Synthesis of TAT-conjugated BSA-FITC.** BSA-FITC (5 mg/mL, 0.075 mM) was dissolved in PBS buffer and the solution was added with SMCC at a SMCC/protein molar ratio of 6:1. The mixture was stirred at dark for 24 h, and then TAT with an N-terminal cysteine was added at TAT/protein molar ratios of 2:1; 4:1 and 6:1, respectively. The reaction solutions were further stirred at dark for 24 h, and the products were dialyzed against PBS (MWCO 3500 Da) and lyophilized before use.

**Preparation of amphiphile/protein nanocomplexes.** The synthesized amphiphiles were mixed with protein in deionized water at different weight ratios for 2 h. The size and zeta potential of the formed nanocomplexes were measured by Zetasizer Nano ZS (Malvern Instrument). The morphology of representative nanocomplexes was observed by transmission electron microscope (TEM, HT7700, HITACHI, Japan). The element mapping was conducted by Field-emission High Resolution Transmission Electron Microscope (Talos F200X, America). The molar ratio of BSA and amphiphiles was 1: 1 in DLS and TEM studies. The concentration of BSA-Pt and amphiphiles was 0.75 μM in element mapping study.

**Cell culture.** HeLa cells (a human cervical carcinoma cell line, ATCC), HEK293 cells (a human embryonic kidney cell line, ATCC), and NIH3T3 cells (mouse embryo fibroblasts, ATCC) were cultured in Dulbecco's modified Eagle's medium (DMEM, GIBCO) containing 100 units/mL penicillin sulfate and streptomycin, and 10% (v/v) fetal bovine serum (FBS, Gemini) at 37 °C in a 5% $CO_2$ atmosphere. The cells were cultured in 48-well plates overnight before protein delivery.

**Cellular uptake of amphiphiles.** The materials F4-1, F3-3, A4-1 and A3-3 were labeled with FITC and then quantitatively analyzed by fluorescence spectroscopy. The samples showed similar fluorescence intensity at an equal polymer mass concentration. FITC-labeled materials diluted with serum-free media were added to the cells cultured in a 48-well plate (5 μg/mL). After incubation for 0.5 h, 1 h, 2 h and 4 h respectively, the culture media were removed and the cells were washed with PBS for three times. Trypan blue was added (0.2 mg/mL) before the cells were quantitatively analyzed by flow cytometry.

To investigate the endocytosis pathways of amphiphile/BSA-FITC complexes, the cells were incubated with endocytosis inhibitors including genistein (700 μM), chlorpromazine (20 μM), cytochalasin-D (10 μM) and sodium azide (100 mM) or treated at 4 °C for 1 h before the addition of amphiphile/BSA-FITC complexes. The untreated cells were tested as controls. The transfection experiments were then conducted as described above, and trypan blue (0.2 mg/mL) was added to the transfected cells before flow cytometry measurement.

**β-Gal activity of the transfected cells.** The β-Gal enzymatic activity of the transfected cells was tested by a β-Gal staining kit and a β-Gal assay kit according to the manufacture's protocols. Generally, HeLa cells were treated with fluoroamphiphile/β-Gal nanocomplexes for 4 h as described above. The cells were then washed with PBS for three times, fixed for 15 min, and further washed with PBS. After that, the transfected cells were incubated with a working solution containing 5% 5-bromo-4-chloro-3-indolyl-β-d-galactopyranoside (X-gal) overnight at 37 °C. The cells were then washed with PBS and observed by an optical microscope (Olympus, Japan). The β-Gal activity in the transfected cells were

quantitatively analyzed by a β-galactosidase assay kit according to the manufacture's protocol (Beyotime Biotech.). Generally, 50 μL of the cell lysates were mixed with 50 μL working solution containing the enzyme substrate O-nitrophenyl-β-D-galactopyranoside, and then the plate was incubated at 37 °C for 30 min. After that, the mixture solution in each well was added with 150 μL stop reaction solution, and the optical density of solutions in the wells at 420 nm was measured using a microplate reader (Thermo Scientific, Germany). The activity of free β-Gal solution at an equal enzyme concentration was tested and considered as 100% relative β-Gal activity. Three repeats were conducted for each sample. For *in vitro* β-Gal activity assay, 2 μg fluoroamphiphiles were incubated with 1 μg, 2 μg or 4 μg β-Gal, respectively in 50 μL PBS buffer for 4 h. After that, the complex solutions were incubated with 50 μL working solution containing 5% X-gal at room temperature for 1 h. 100 μL DMSO was added to dissolve the yielding product and the optical density of solution at 633 nm was measured using a microplate reader. Free β-Gal solutions in the absence of fluoroamphiphiles were tested as controls. To confirm that the β-Gal activity in the complexes can be recovered after cytosolic delivery, the complex solutions were also incubated with heparin sodium (0.1 mg/mL) for 1 h, and then the β-Gal activity was measured as described above.

**MTT assay.** The cytotoxicity of amphiphiles or amphiphile/protein nanocomplexes was measured by a well-established MTT assay. Generally, HeLa cells were seeded in a 96-well plate at a density around $10^4$ cells per well overnight. The cells were incubated with the amphiphiles, amphiphile/BSA-FITC nanocomplexes (5 μg/mL to 15 μg/mL polymer, 20 μg/mL BSA) for 4 h. After that, the incubation media were removed and the cells were further incubated with fresh DMEM containing 10% FBS for 20 h. Then a standard MTT assay was used to determine the cytotoxicity. Five repeats were conducted for each sample. For cytosolic saporin toxicity assay, the saporin concentration ranges from 0-33 nM and the polymer concentration was fixed at 0.28 μM, respectively.

**Circular dichroism analysis of nanocomplexes.** F4-1, F3-3, A4-1, A3-3 and unmodified PEI were mixed with BSA (100 μg) in 500 μL deionized water at a molar ratio of 1: 1 for 4 h. The final protein concentration is 200 μg/mL (3 μM). Free BSA solution was tested as a control. Then the nanocomplex solutions were analyzed by circular dichroism spectrometer (CD/J-815, Japan).

**BCA protein assay.** Fluoroamphiphiles were mixed with BSA (40 μg) in PBS at a molar ratio of 1: 1 for 4 h. Then the protein concentration was tested by a BCA protein assay kit according to the manufacture's protocol. Generally, reagent A and reagent B at a volume ratio of 1: 50 were mixed before BCA assay. Then the working solution was pipetted into microplate wells (100 μL/well) and added with the prepared sample. After that, the wells were thoroughly mixed and incubated at room temperature for 45 min. Finally, absorbance of the solution at 562 nm was measured by a plate reader (Thermo Scientific, Germany). A standard curve was made and the concentration of sample protein was calculated according to the curve.
